# Phosphorus transformations and leaching potential in rewetting drained peatlands: Exploring the influence of land use and temperature

**DOI:** 10.1007/s10653-025-02751-y

**Published:** 2025-09-17

**Authors:** Atif Muhmood, Haonan Guo, Lorenzo Pugliese, Shubiao Wu

**Affiliations:** 1 Department of Agroecology, Aarhus University, Blichers Alle 20, 8830 Tjele, Denmark; 2 Institute of Soil Chemistry and Environmental Sciences, AARI, Faisalabad, Pakistan

**Keywords:** Peatlands, Land use, Temperature, Rewetting, Phosphorus dynamics, Climate change

## Abstract

Understanding phosphorus (P) transformation dynamics during peatland rewetting is crucial for developing effective management strategies, supporting ecological restoration initiatives and mitigating potential environmental risks. This incubation study explored the temporal variations in P transformation in peatlands under different land uses (cut grass, grazing, unmanaged) along with the potential risk of leaching by simulating rewetting conditions for four months at varying temperatures (10 and 20 °C). Overall, only a small fraction of the initial total phosphorus (P) was leached during rewetting. Among land uses, soils under grazing showed the highest release (0.34%), followed by cut grass (0.19%) and unmanaged land (0.13%). A higher risk of leaching at the beginning of the rewetting was observed to be associated with a high transformation rate of P from organic form to inorganic form but mitigated by resorption with ongoing rewetting. Soil organic carbon, initial contents of P, iron, and aluminium as well as temperature were found to be the main factors controlling P transformation and leaching during the rewetting process. Increasing incubation temperature from 10 °C to 20 °C raised P release by 33–41%. Moreover, a projected 1.5 °C increase in temperature due to climate change is estimated to increase the rate of phosphorus (P) transformation and release by approximately 24%, based on temperature sensitivity analysis. More research is needed to comprehensively explore complex interactions involving seasonal variations, microbial activity, and geological processes. This is also necessary for a holistic understanding of how these ecosystems may respond to ongoing climate changes.

**Supplementary Information:**

The online version contains supplementary material available at 10.1007/s10653-025-02751-y.

## Introduction

Peatlands cover less than 3% of the Earth's land surface yet store around one-third of the world's terrestrial soil organic carbon and serve as vital sources of freshwater (Xu et al., 2018). Additionally, these unique ecosystems are home to fragile flora and fauna species, contributing significantly to nature conservation efforts (Renou-Wilson, 2018). Unfortunately, extensive drainage for agricultural purposes has transformed many peatlands from carbon sinks to sources, impairing their capacity to regulate water quality and causing biodiversity loss (Kreyling et al., 2021). Given these alarming trends, it is imperative to prioritize the restoration of degraded peatlands. Thus, rewetting degraded peatlands by raising the water table is essential to reinstate their carbon-sequestrating function and to promote the recolonization of peat-forming plant communities (Andersen et al., 2013).

Despite the importance of peatland restoration, phosphorus (P) leaching presents a significant challenge during the rewetting process due to its potential to contribute to downstream eutrophication (Pönisch et al., 2023). Significant P leaching has been observed during the rewetting of certain drained peatlands, both through field and laboratory investigations (Forsmann & Kjaergaard, 2014; Harpenslager et al., 2015; Zak et al., 2017). However, it's crucial to recognize that not all rewetted peatlands exhibit substantial P leaching (Florea et al., 2024; Meissner et al., 2008, 2010). Consequently, the current state of knowledge remains fragmented, making it challenging to draw definitive conclusions regarding the specific areas where P leaching may occur upon rewetting. Despite this uncertainty, it is imperative to guide policymakers and expedite rewetting efforts, given the potential environmental implications associated with P leaching.

The mechanism of P retention and leaching during the rewetting of drained peatlands remains inadequately understood, owing to its complexity and dependence on various factors such as prior land use, soil texture and composition, and management practices (Kaila et al., 2016; Zak et al., 2010). Intensive farming and the application of fertilizers have been observed to cause the buildup of P in soils, increasing the risk of leaching upon rewetting (Nest et al., 2015). Normally, P becomes extensively bound in the surface soil through redox-sensitive complexes of Fe, Al and Ca. These complexes subsequently dissolve when the soil is saturated with water, triggering the reduction of ions upon rewetting, which in turn may release P into the porewater (Curtinrich et al., 2022; Nieminen et al., 2020). Moreover, in rewetted peatlands, the mineralization of organic P into labile fractions might be facilitated. Simultaneously, the degradation of particulate organic matter would function as an auxiliary mechanism for P mobilization during the rewetting process (Guo et al., 2022). Therefore, understanding the complex interplay of P transformations within rewetted peatlands and finding out the main driving factors hold the key to mitigating the potential risk of leaching, thus safeguarding water quality for future generations.

In the ever-evolving narrative of climate change, an escalation in the frequency, severity, and duration of extreme weather events is witnessed, exemplified by the rise of prolonged droughts and scorching heat waves, leading to transient high temperatures (Cook et al., 2014; Diffenbaugh et al., 2017). Elevated temperatures have the potential to influence the dynamics of P transformations by accelerating organic matter decomposition and stimulating phosphatase activities (Guo et al., 2021; Li et al., 2019). Additionally, rising temperatures may exacerbate sub-surface P losses, as the escalation of desorption mechanisms tends to outpace sorption processes, intensifying the vulnerability of P dynamics within the system (Hanyabui et al., 2020). Nevertheless, in the context of peatlands rewetting, the influence of projected temperature changes under future climates on P dynamics and its potential loss in soluble forms remains uncertain. Therefore, understanding the interactions between climate change and P dynamics is crucial for implementing effective management strategies to mitigate potential environmental risks associated with P leaching in rewetted peatlands.

To address these knowledge gaps, we conducted a comprehensive sampling effort across two distinct river catchments, each characterized by three representative land uses (cut grass—CG, grazing—GR, and unmanaged/natural—UM). These land-use types reflect common management practices in Danish peatlands and collectively represent a broad spectrum of land-use intensity—from actively managed systems (cut and grazed) to passive, unmanaged areas. Studying this range offers a comprehensive understanding of how different management regimes influence phosphorus dynamics during rewetting (Liu et al., 2018). Moreover, it enables the assessment of how legacy phosphorus (P) stocks and their transformations vary with land use during rewetting. Managed systems, such as grazed and cut grasslands, may respond differently to anoxic conditions and rising temperatures compared to unmanaged lands, which often exhibit distinct P release kinetics due to higher organic matter accumulation and altered redox dynamics (Fu et al., 2021). This extensive sampling enabled us to explore the intricate dynamics of P transformation within organic soils exhibiting a wide range of organic carbon (OC), P, Fe and Al contents. Through batch incubation experiments simulating rewetting, P transformation dynamics in topsoil samples were investigated for four months and at varying temperatures (10 °C and 20 °C). The selected temperatures represent realistic environmental conditions: 10 °C reflects the average peat soil temperature in Denmark during spring and autumn, serving as a baseline, while 20 °C simulates warmer summer conditions or projected future scenarios under climate change (Lembrechts et al., 2020). This temperature range enables the investigation of how warming affects phosphorus dynamics, offering valuable insights into potential climate-induced shifts in nutrient release during peatland rewetting. The aims of the study were to (i) discern where and when to strategically implement rewetting initiatives, (ii) how P transformation dynamics respond to different land uses and rewetting temperatures, and (iii) evaluate if P leaching warrants serious consideration. Our findings provide valuable insights for the strategic implementation of rewetting initiatives and highlight the importance of considering P dynamics in peatland restoration efforts.

## Materials and methods

### Soil sampling and processing

**Fig. 1:**
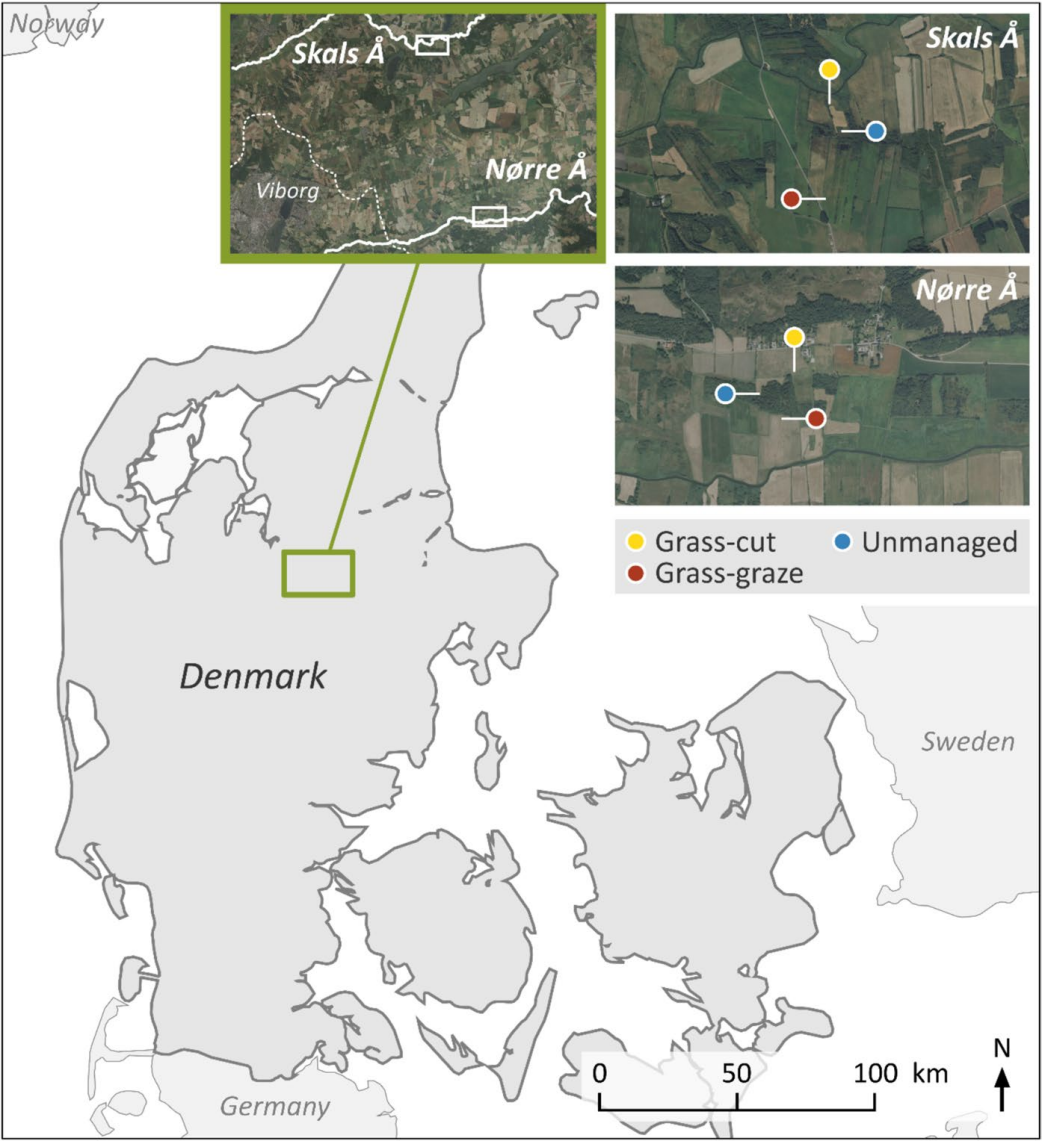
Map showing sample collection locations

The study area, situated in central Jutland, experiences a temperature range from 1 °C in February to 17 °C in July, with annual precipitation ranging between 650 and 875 mm. In February 2023, peat soil samples were collected from two distinct river catchments at Skals and Nørre (Fig. 1). At each site, undisturbed soil core samples (8.5 × 6.0 cm) were systematically collected in triplicate from the upper layer of organic soils subject to different land uses, namely CG, GR and UM. A total of 36 core samples were collected, consisting of two sets of 18 samples each [3 land uses × 2 locations × 3 replications], to evaluate phosphorus (P) transformation under simulated rewetting conditions at two distinct temperatures: 10 °C and 20 °C. At Skals, samples were taken from CG (56.54864°N, 9.57328°E), GR (56.54414°N, 9.57305°E), and UM (56.54740°N, 9.57430°E). At Nørre, corresponding samples were collected from CG (56.45252°N, 9.63143°E), GR (56.45088°N, 9.63024°E), and UM (56.45184°N, 9.62863°E). The collected samples were transported to the laboratory with due care, and subsequently stored at 4 °C until their use in the incubation study. Concurrently, soil samples were randomly collected from each sampling location and combined into a composite sample (approximately 1.0 to 1.5 kg). This composite sample underwent drying at 60 °C for 48 h, followed by sieving through a 2 mm mesh and subsequently used for the analysis of the soil chemical properties.

### Batch incubation experimental setup

An incubation study was undertaken to investigate the impact of rewetting on drained peatlands under varying land uses, with a specific focus on the dynamics of P transformation into inorganic and organic fractions. Incubation studies were conducted in dark conditions to mimic subsurface soil environments, minimize photochemical reactions, and prevent algal or microbial photosynthetic activity that could alter P dynamics. Soil cores were placed within glass jars (16 × 10 cm), and rewetting conditions were simulated by maintaining a water level of 5 cm above the surface of the soil core. The jars were closed with airtight lids. To evaluate the temperature-dependent effects on P transformation during the rewetting process, the jars were incubated at two distinct temperatures, namely 10 °C and 20 °C in different rooms for a duration of four months. Approximately 10 ml of water sample was collected from the topsoil core in each jar using a syringe while around 5–10 g soil sample was taken using a spoon from each core in the jar. Water and soil samples were collected at one-month intervals in triplicate for the analysis of soluble phosphate, as well as various inorganic and organic P fractions to ensure statistically reliable results.

### Analytical methods

#### Soil analysis

Soil pH was measured by immersing 5 g of dry soil in 50 mL H_2_O overnight, employing a PHM210 Standard pH Meter (Radiometer Analytica, Brønshøj, Denmark). Organic carbon (OC) in soils were analyzed by dry combustion at 950 °C using a Vario Max Cube (Elementar Analysensysteme GmbH, Hanau, Germany). The soil samples were devoid of carbonates (effervescence test) and total carbon was considered OC. Total contents of P, Al, Fe and Ca in soils were determined by Thermo Scientific—iCAP 6000 Series ICP-OES after digesting 0.1 g ball-milled dry soil in a mixture of 1 ml concentrated H_2_SO_4_ and 2 ml concentrated HClO_4_ for one hour at 250 °C on a Tecator digestion unit (FOSS A/S, Hillerød, Denmark). Results are reported on a soil dry mass (DM) basis.

#### Sequential P analysis

**Fig. 2:**
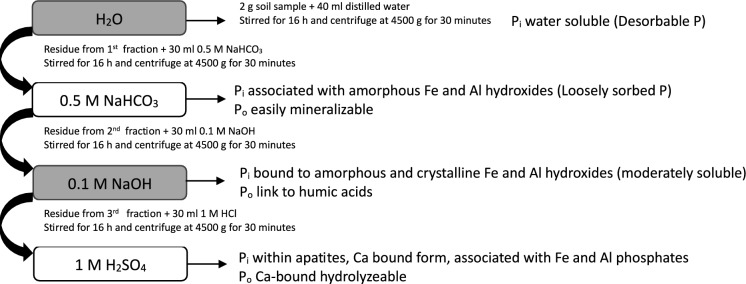
Scheme of sequential phosphorus (P) analysis process

Soil samples underwent sequential fractionation utilizing the modified Hedley P fractionation method (Graça et al., 2022; Negassa et al., 2020). This protocol allowed fractionation into four inorganic P (P_i_) fractions (H_2_O.P_i_, NaHCO_3_.P_i_, NaOH.P_i_, and H_2_SO_4_.P_i_) and three organic P (P_o_) fractions (NaHCO_3_.P_o_, NaOH.P_o_, and H_2_SO_4_.P_o_). The H_2_O.P fraction represented desorbable P while NaHCO_3_ extracted P represented loosely sorbed bioavailable P_i_ linked to Al and Fe oxides, as well as easily mineralizable P_o_. The NaOH.P fraction included P_i_ associated with amorphous and crystalline Fe and Al hydroxides, clay minerals, and P_o_ components associated with humic substances. The H_2_SO_4_.P fraction represents P within apatite minerals and calcium phosphate forms, associated with Fe and Al phosphates occluded within sesquioxides, and P_o_ related to Ca-bound hydrolysable P_o_ (Pätzold et al., 2013; Velásquez et al., 2016). The organic P content in all P fractions (NaHCO_3_.P_o_, NaOH.P_o_, and H_2_SO_4_.P_o_) was computed as the difference between total P (P_t_) and P_i_ for each fraction. In terms of labile and non-labile forms, H_2_O.P_i_ and NaHCO_3_.P_i_ is considered as labile P while NaOH.P_i_, and H_2_SO_4_.P_i_ belongs to non-labile P. A schematic representation of the sequential P fractionation process is illustrated in Fig. 2.

For P fractionation, 2 g of soil was carefully weighed and placed into 50 mL centrifuge plastic tubes. The extraction process involved adding 40 mL of distilled water and subjecting the tubes to 16 h of stirring on an end-over-end shaker. Following this step, the samples underwent centrifugation at 4500 g for 30 min, after which the resultant mixture was carefully filtered. The residual soil was subjected to an extraction process performed by using 30 mL of 0.5 M NaHCO_3_ (pH 8.5) for an additional 16 h. The supernatant was then collected using the earlier-mentioned method. Further chemical fractionation of the remaining soil was conducted with 30 mL of 0.1 M NaOH and 1 M H_2_SO_4_ successively. At the end of each fractionation, the supernatant was collected. The Pi concentration in all extracts was measured utilizing the molybdenum blue method (Murphy & Riley, 1962) employing a Thermo Spectronic Helios Alpha 9423 UVA 1002E UV–VIS Spectrophotometer (Thermo Fisher Scientific, Waltham, MA, USA).

The total P concentration in each fraction was determined by digesting the extract with sulfuric acid (H_2_SO_4_) and ammonium persulfate [(NH_4_)_2_S_2_O_8_]. The procedure involved adding 1 mL of 11 N sulfuric acid solution (prepared by adding 28 mL of concentrated H_2_SO_4_ in 100 mL) to a 50 mL sample in a 125 mL Erlenmeyer flask. Subsequently, 0.4 g of ammonium persulfate was added, and the mixture gently boiled on a pre-heated hot plate for approximately 30–40 min until the volume was reduced to around 10 mL. Caution was taken to prevent the sample from drying out. The sample was then allowed to cool, and if necessary, it was filtered to ensure clarity. Finally, the sample was diluted to 50 mL, and the P content was determined using the molybdenum blue method (Murphy & Riley, 1962).

#### Temperature sensitivity quotient

The Temperature Sensitivity Quotient (Q_10_) serves as a measure of the relative change in a process with temperature variation. It is defined as the ratio of the rate of a process at a given temperature to the rate of the same process at a reference temperature. Q_10_ was calculated using the following equation (Ito et al., 2015):

1 $${\text{Q}}_{10} = \;\left[ {\frac{{{\text{R}}2}}{{{\text{R}}1}}} \right]^{{\left( {\frac{10}{{{\text{T}}2 - {\text{T}}1}}} \right)}}$$

where Q_10_ is the temperature sensitivity constant, R_2_ and R_1_ are the reaction rate at temperatures T_2_ and T_1_, respectively.

#### Statistical analysis

Structural Equation Modeling (SEM) was employed to investigate the influence of various factors including soil pH, soil OC, initial P content and temperature on P transformations into different fractions. SEM integrates factor analysis and path analysis to examine relationships among variables (Chen et al., 2023). The analysis was executed utilizing IBM SPSS AMOS 24 software. To comprehensively evaluate the model fit, a combination of widely accepted fit indices was employed, including the Chi-square statistic (CMIN), the relative Chi-square (CMIN/DF), Goodness-of-Fit Index (GFI), Adjusted Goodness-of-Fit Index (AGFI), Tucker-Lewis Index (TLI), and Root Mean Square Error of Approximation (RMSEA). Model fit was considered acceptable based on the following threshold criteria: CMIN/DF ≤ 3 (or ≤ 5 in less stringent contexts), GFI, CFI, and TLI ≥ 0.90, and RMSEA ≤ 0.08. Moreover, one-way analysis of variance (ANOVA) was used to evaluate significant differences between different land use for the P contents in various fractions, with a level of significance set at p < 0.05. Sigma plot software (version 12.5, Systat Software Inc., San Jose, CA, USA) and Origin Pro 2023 were used for data analysis and plotting.

## Results

### Soil characterization

The characteristics of soils under different land uses were highly variable (Table 1). Soil pH varied from extremely acidic (pH 3.90) to slightly acidic (pH 6.24), with the majority of samples showing very strong acidity. Similarly, the content of soil total organic carbon ranged from 10.7 to 38.7% of dry mass, with nearly all soils classified as peat (> 12% OC), except for the GR sample at Skals. Soil bulk density ranged from 0.26 to 0.64 g cm^−3^, while TN ranged from 0.80 to 2.73%. Ammonium nitrogen (NH_4_-N) concentrations ranged between 0.87 and 9.98 mg kg^−1^ and NO_3_-N varied from 1.14 to 15.8 mg kg^−1^. The total P and Fe exhibited wider ranges of variation from 27.2 to 159 mmol kg^−1^ and from 64.5 to 2103 mmol kg^−1^, respectively, across soils under various land uses. Furthermore, the contents of total Al and Ca varied from 48.2 to 198 mmol kg^−1^ and from 67.9 to 307 mmol kg^−1^, respectively.

**Table 1:** Properties of topsoil samples from two river catchments subject to different land uses. Standard deviations are also given

River catchment	Land use and coordinates	pH-	TOC Mass %	BD g cm^**−**3^	TP mmol kg^**−**1^	Fe mmol kg^**−**1^	Al mmol kg^**−**1^	Ca mmol kg^**−**1^
Skals	CG (56.54864°N, 9.57328°E)	5.23 ± 0.05	30.8 ± 1.77	0.42 ± 0.03	151 ± 9.6	2103 ± 213	117 ± 8.73	164 ± 21.4
	GR (56.54414°N, 9.57305°E)	6.24 ± 0.02	10.7 ± 1.21	0.64 ± 0.07	37.2 ± 3.4	80.2 ± 15.7	198 ± 17.6	136 ± 16.7
	UM (56.54740°N, 9.57430°E)	3.90 ± 0.07	36.1 ± 0.43	0.38 ± 0.01	159 ± 18.3	858 ± 86.5	48.2 ± 3.12	73.7 ± 9.62
Nørre	CG (56.45252°N, 9.63143°E)	5.03 ± 0.08	28.4 ± 3.56	0.41 ± 0.09	38.1 ± 3.9	117 ± 22.9	174 ± 20.7	146 ± 24.7
	GR (56.45088°N, 9.63024°E)	4.92 ± 0.03	20.5 ± 0.46	0.44 ± 0.04	27.2 ± 3.0	99.4 ± 13.8	121 ± 14.2	67.9 ± 10.1
	UM (56.45184°N, 9.62863°E)	4.78 ± 0.08	38.7 ± 1.75	0.26 ± 0.02	28.2 ± 1.3	64.5 ± 3.24	82.1 ± 3.10	307 ± 7.95

*TOC* total organic carbon, *BD* bulk density, *TN* total nitrogen, *NH*_*4*_*-N* ammonium nitrogen, *NO*_*3*_^*−*^*–N* nitrate nitrogen, *TP* total P, *Fe* iron, *Al* aluminium, *Ca* calcium

### P fractions before rewetting

**Fig. 3:**
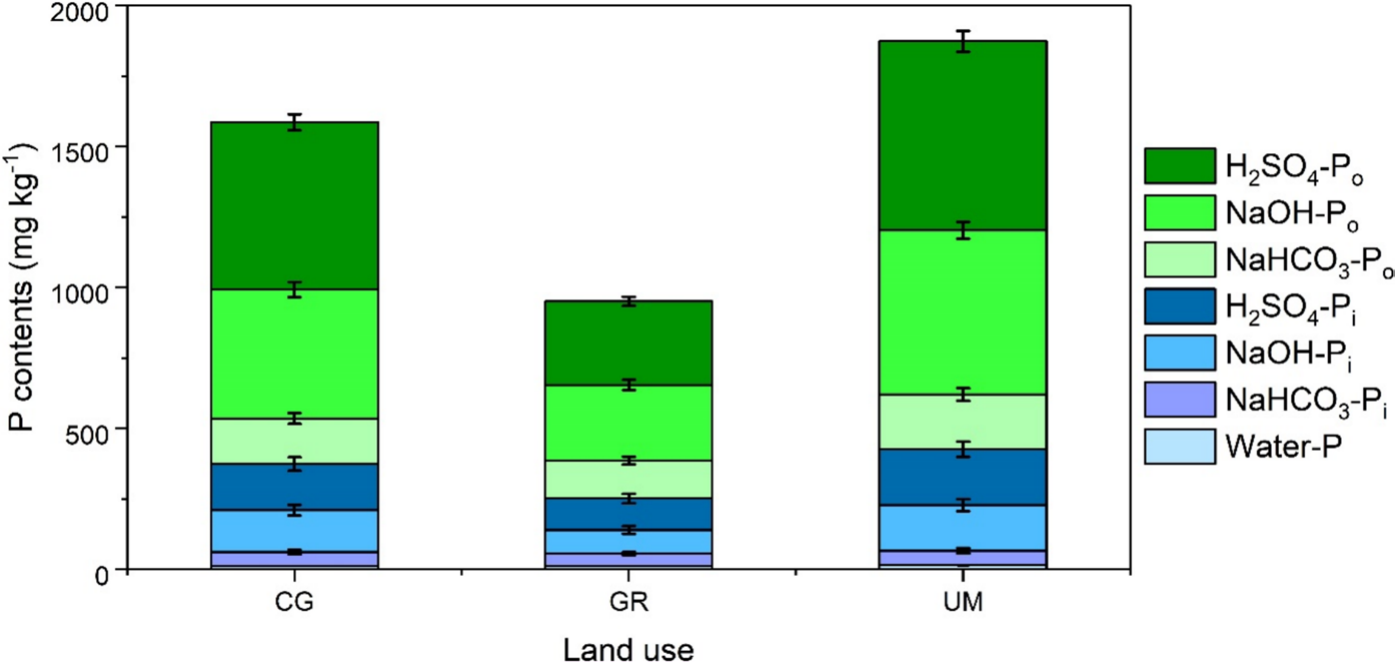
Contents of P in different fractions in peatlands from different land uses (CG = cut grass, GR = grazing, UM = unmanaged) (Error bars represent ± SD)

Organic P (P_o_) was the predominant component of total P (P_t_), ranging from 681 ± 155 to 1879 ± 1303 mg kg^−1^ (74–78% of P_t_) (Fig. 3, Table S1). When comparing different land uses, soils under GR had statistically lower P_o_ contents compared with CG and UM (*p* < 0.05). A detailed examination of individual fractions revealed that the contents of P_o_ fractions extracted using bicarbonate (NaHCO_3_), alkali (NaOH), and acid (H_2_SO_4_) ranged from 133 ± 33 to 225 ± 68, 244 ± 47 to 786 ± 611 and 303 ± 89 to 867 ± 624 mg kg^−1^, respectively, across soil under various land uses.

Furthermore, inorganic P (P_i_) content in soils under different land uses ranged from 229 ± 34 to 519 ± 333 mg kg^−1^ (around 22–26% of P_t_). Considering different P_i_ fractions, the average contents of water, bicarbonate, alkali, and acid-extracted P ranged from 11.4 ± 2.62 to 14.9 ± 3.40, 38.5 ± 7.86 to 59.8 ± 14.4, 71.4 ± 20.0 to 211 ± 159, and 109 ± 14.3 to 232 ± 160 mg kg^−1^, respectively. Statistically significant differences in the contents of different P_i_ fractions were observed in soils under different land uses (*p* < 0.05).

### P fraction under rewetting

**Fig. 4:**
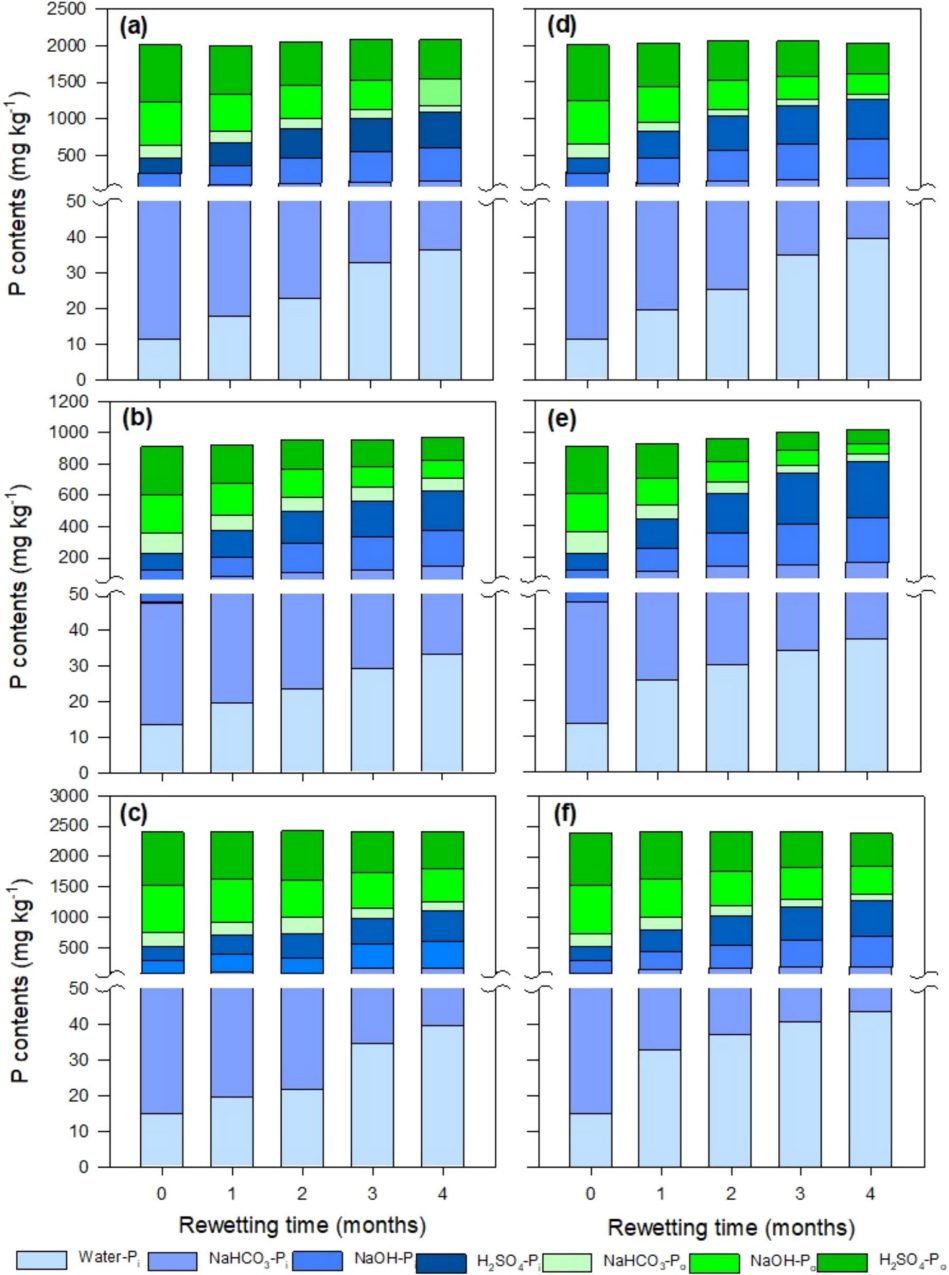
Contents of P in different inorganic (P_i_) and organic (P_o_) fractions in soils under various land uses during rewetting at 10 °C (a = cut grass, b = grazing, c = unmanaged) and 20 °C (d = cut grass, e = grazing, f = unmanaged)

The proportion of P_o_ under rewetting decreased, ranging from 1879 ± 1303 to 335 ± 132 mg kg^−1^ (a decrease in proportion from 78 to 35%) irrespective of the incubation temperature with incremented rewetting time from 1 to 4 months (Fig. 4, Tables S1 and S2). A continuous decline in individual P_o_ fractions in soil under various land uses was observed with increasing rewetting time, ranging from 225 ± 68 to 77 ± 28, 786 ± 611 to 137 ± 48, and 867 ± 624 to 165 ± 61 mg kg^−1^ for bicarbonate, alkali, and acid extractable, respectively.

**Fig. 5:**
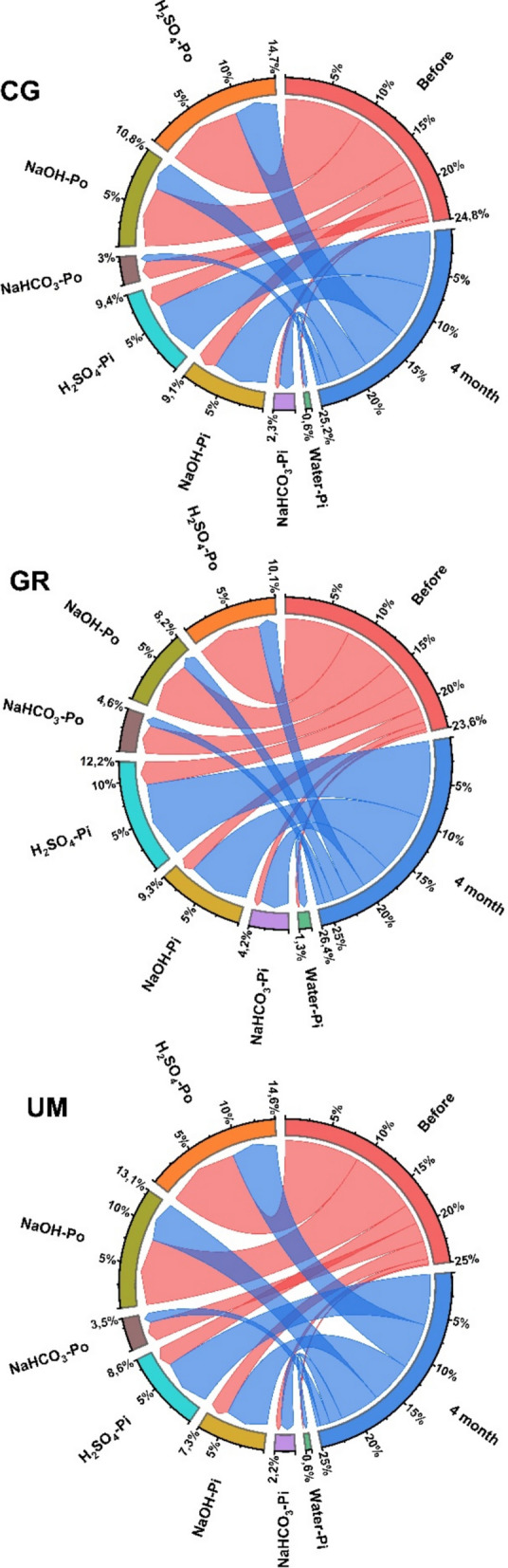
Trend of change in the contents of P in different fractions in soils under various land uses (CG = cut grass, GR = grazing, UM = unmanaged) after four months of rewetting 20 °C

In contrast, P_i_ content increased from 229 ± 34 to 993 ± 503 mg kg^−1^ (an increase in proportion from 22 to 66%) with incremented rewetting time from 1 to 4 months, irrespective of incubation temperature (Fig. 4). Likewise, individual P_i_ fractions increased, ranging from 11.4 ± 2.62 to 39.2 ± 6.75, 38.5 ± 7.86 to 119 ± 20.5, 71.4 ± 20.0 to 410 ± 150 and 109 ± 14.3 to 449 ± 170 mg kg^−1^ for water, bicarbonate, alkali, and acid extracted fractions, respectively, at varying rewetting time. Furthermore, all land uses were statistically significant with each other for variations in P_o_ and P_i_ fractions during the rewetting process (*p* < 0.05). A trend of variations in different fractions (P_o_ and P_i_) before and after rewetting is depicted in (Fig. 5 and Fig. S1).

**Fig. 6:**
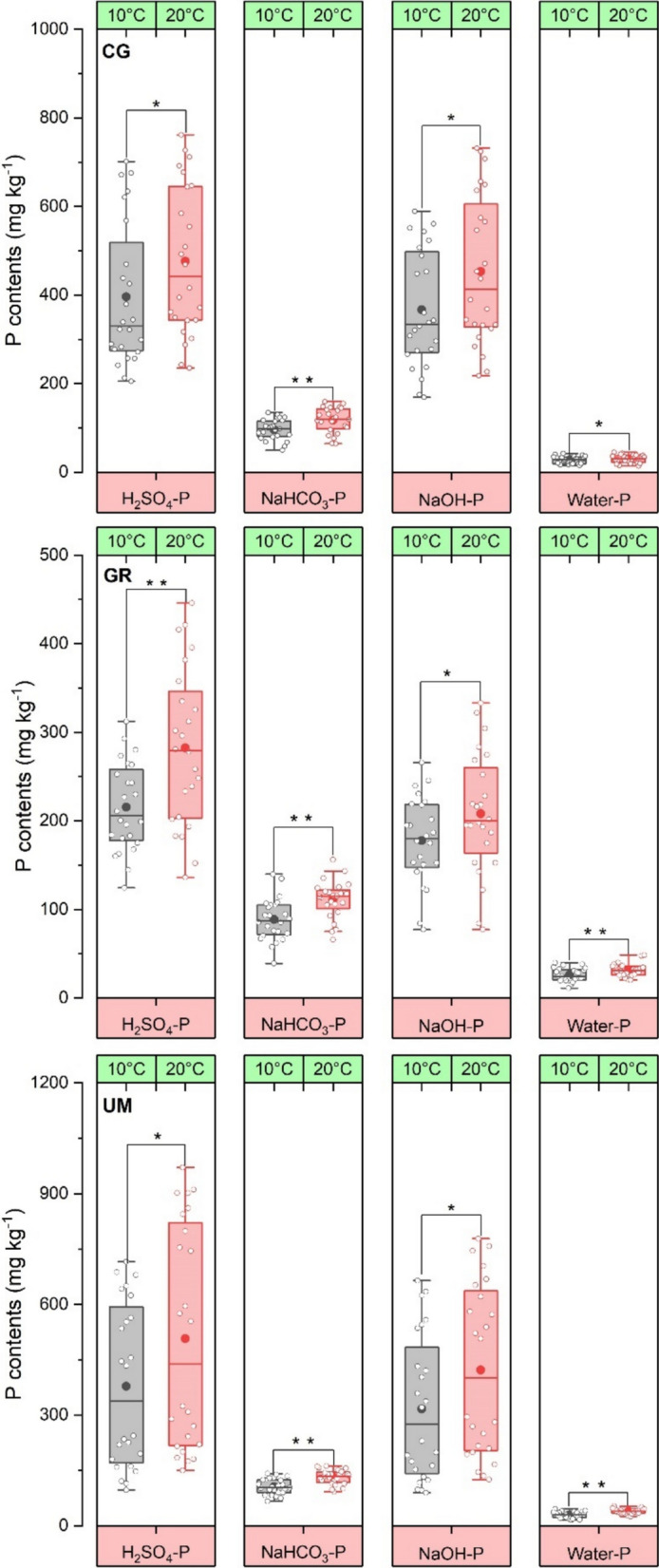
Variation in the contents of inorganic P fractions in soils under various land uses (CG = cut grass, GR = grazing, UM = unmanaged) after four months of rewetting under varying temperature

Significant variations in different fractions of P (P_i_ and P_o_) were also observed under varying rewetting temperatures (Fig. 6 and Fig. S2). On an overall average basis, P_o_ fractions decreased from 128 ± 43 to 101 ± 41, 405 ± 270 to 343 ± 249, and 493 ± 365 to 428 ± 316 mg kg^−1^ for bicarbonate, alkali, and acid extractable P, respectively, with increasing rewetting temperature from 10 to 20 °C. In contrast, P_i_ fractions increased from 27 ± 7.71 to 34 ± 8.20, 96 ± 22 to 120 ± 23, 306 ± 156 to 356 ± 134, and 346 ± 150 to 420 ± 180 mg kg^−1^ for water, bicarbonate, alkali, and acid extractable P, respectively, with an increase in rewetting temperature from 10 to 20 °C. Significant differences were found among various land uses for the variations in the contents of different P fractions at varying rewetting temperatures (*p* < 0.05).

**Fig. 7:**
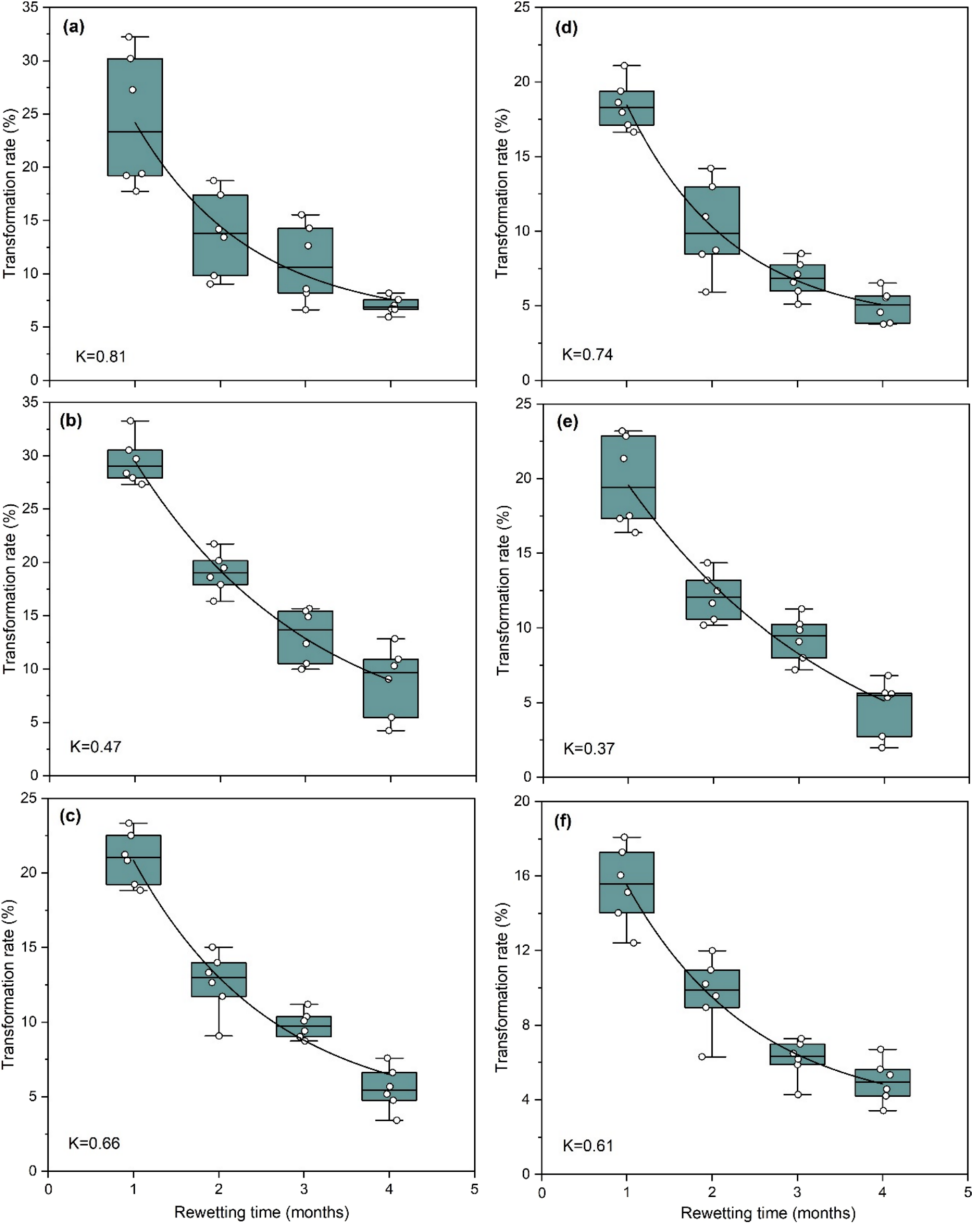
Transformation rate of organic P during rewetting of soil under different land uses at 20 °C (a = cut grass, b = grazing, c = unmanaged) and 10 °C (d = cut grass, e = grazing, f = unmanaged)

The Q_10_ results revealed that the average values ranged from 1.09 ± 0.08 to 1.19 ± 0.10, 1.14 ± 0.10 to 1.29 ± 0.13, 1.06 ± 0.08 to 1.37 ± 0.11 and 1.20 ± 0.13 to 1.58 ± 0.11 for the change in water, bicarbonate, alkali, and acid extractable P_i_ fractions, respectively (Fig. 7). Meanwhile, Q_10_ values for different P_o_ fractions ranged from 1.18 ± 0.10 to 1.35 ± 0.12, 1.15 ± 0.11 to 1.20 ± 0.10, and 1.10 ± 0.09 to 1.21 ± 0.10 for bicarbonate, alkali and acid extractable P_o_ fractions, respectively after rewetting soils.

### P release in water during rewetting

**Fig. 8:**
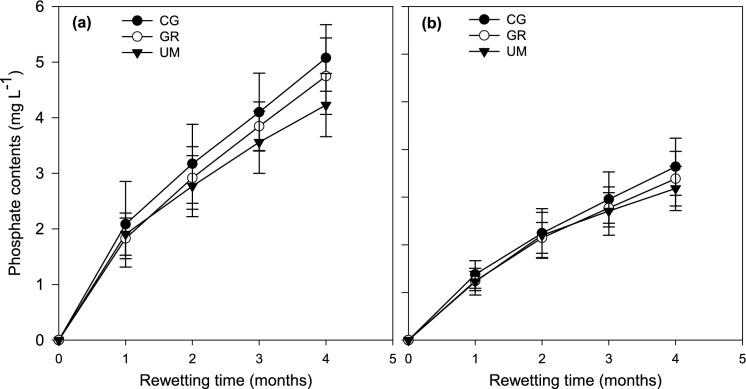
Variation in the P contents in water during rewetting of soils under various land uses (CG = cut grass, GR = grazing, UM = unmanaged) at 20 °C (**a**) and 10 °C (**b**)

The results regarding the contents of P release in water during the rewetting process showed incremented contents with prolonged rewetting time (Fig. 8). Specifically, the average P contents exhibited increments from 2.08 ± 0.59 to 4.92 ± 0.70, 1.83 ± 0.33 to 4.48 ± 0.69, and 1.90 ± 0.40 to 4.213 ± 0.57 mg L^−1^ in water leached from soils under CG, GR, and UM, respectively, with increasing time during rewetting at 20 °C. In contrast, lower average contents of P ranged from 1.37 ± 0.29 to 3.63 ± 0.60_,_ 1.24 ± 0.20 to 3.28 ± 0.47 and 1.22 ± 0.28 to 2.92 ± 0.46 mg L^−1^ were leached from the soils under CG, GR, and UM, respectively, with incremented rewetting time during rewetting at 10 °C. When looking at the release of P at varying rewetting times, the average contents released in water were around 1.65, 1.00, 0.82 and 0.80 mg L^−1^, 1.60, 0.96, 0.78 and 0.77 mg L^−1^ and 1.57, 0.82, 0.58, 0.57 mg L^−1^ after 1–4 months, for CG, GR and UM respectively.

## Discussion

Peatland drainage for agriculture, forestry, and other land uses profoundly disrupts natural hydrology and alters nutrient cycling, particularly phosphorus (P) dynamics (Guo et al., 2021). Rewetting these systems initiates complex biogeochemical processes, where P transformation is strongly influenced by land-use history, organic matter content, and redox conditions. In the context of climate change, rising temperatures can intensify microbial activity and redox oscillations, potentially enhancing P mobilization and release into surrounding environments (Li et al., 2019). Understanding how P behaves under rewetting across different land uses and temperature regimes is therefore critical for predicting nutrient fluxes and designing peatland restoration strategies that support water quality and ecosystem resilience.

### Re-assessing the risk of P leaching

Peatlands play a crucial role in global ecosystems as substantial carbon reservoirs and unique biodiversity hotspots (Luo et al., 2021). However, the drainage of peatlands for agricultural or other purposes alters their hydrological conditions, resulting in high CO_2_ emissions (Tattari et al., 2017). Therefore, rewetting drained peatlands is becoming an important international strategy to combat the changing climate and reestablish important ecological functions (Sowiński et al., 2024). In this process, P leaching has been recognized as a common concern resulting from increased mobility and availability of P under anaerobic conditions during rewetting (Comber et al., 2023). However, the impact of rewetting on P leaching is contradictory in the sense that high exports generally occur from certain sites, whereas other sites may exhibit low export rates after restoration (Kaila et al., 2016; Koskinen et al., 2011, 2017). Therefore, the knowledge regarding in what conditions and how P leaching in these organic soils upon rewetting can be problematic, and where to rewet, the potential P leaching can be minimized is insufficient and unclear.

**Fig. 9:**
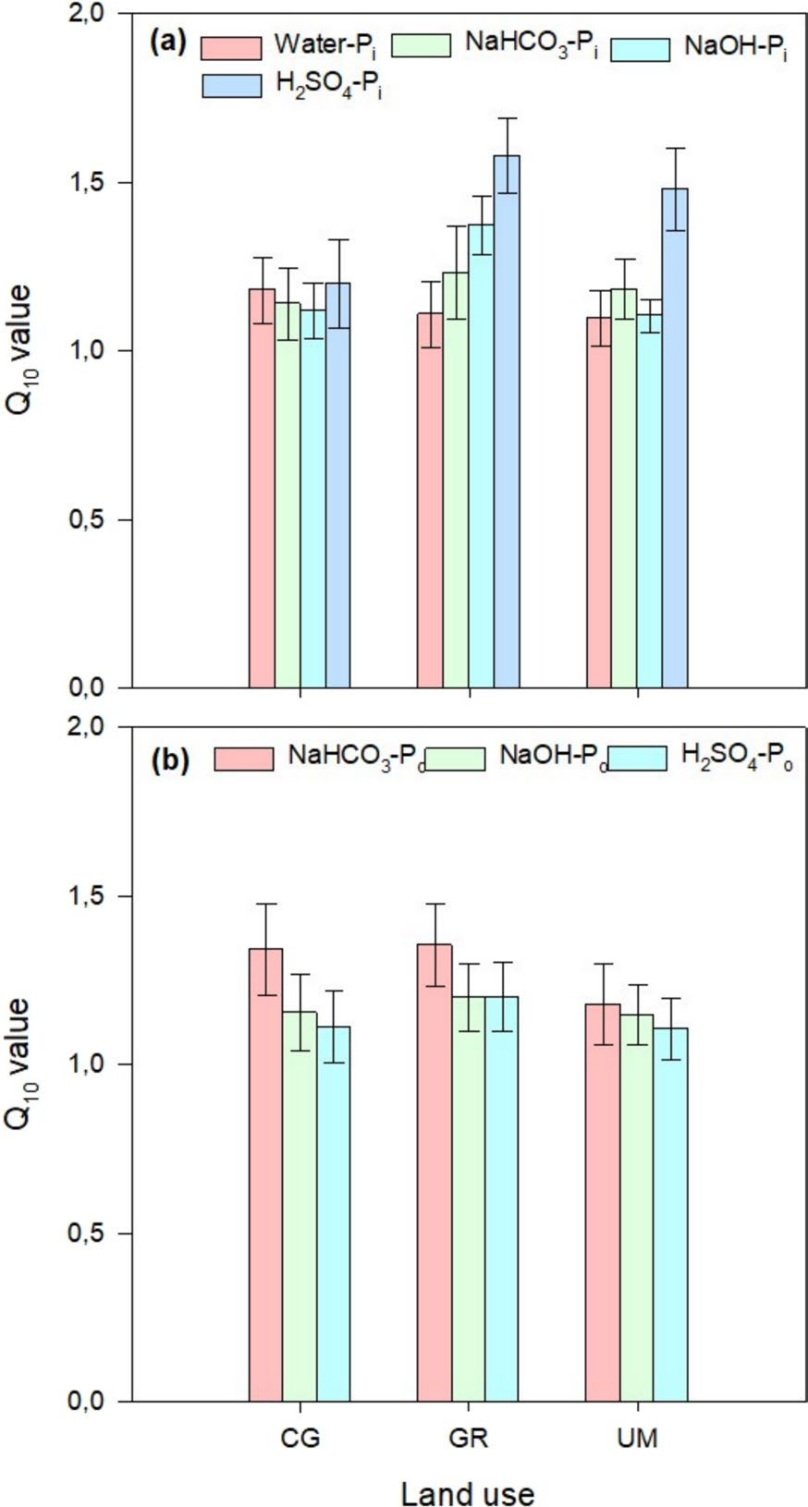
Temperature sensitivity quotient (Q10) values for different phosphorus inorganic (**a**) and organic (**b**) fractions in soils under different land uses (CG = cut grass, GR = grazing, UM = unmanaged) after rewetting

In this study, we also observed P leaching, approximately 0.19%, 0.34% and 0.13% of initial TP, for CG, GR, and UM respectively (Fig. 8). This might be ascribed to the reduction of iron complexes under saturated conditions, as P is usually bound to the surface of highly decomposed peat through redox-sensitive Fe, and Al complexes (Kaila et al., 2016). The differences in the content of leached P among land uses are likewise believed to be attributed to the variations in the contents of Fe and Al (Table 1). The Fe:P molar ratio has been recognized as one of the factors affecting P release and a value of more than 10 is indicative of negligible to no leaching (Forsmann & Kjaergaard, 2014; Zak & Gelbrecht, 2007). Besides, Kaila et al. (2016) reported that the higher initial extractable P contents can also be responsible for the release of P to water. In this study, the Fe:P molar ratio and initial P contents were both found to be responsible for variations in the contents of leached P. The higher contents of P in water observed during rewetting soil under GR were attributed to a lower Fe:P molar ratio (2.90) compared with CG and UM (with Fe:P molar ratio of 8.49 and 3.85 respectively). Moreover, a positive correlation between the initial contents of P and the contents released in water further confirmed the differences in land uses for P leaching (Fig. 9). In addition to chemical factors, land-use-specific management practices also contributed to P losses. Grazing lands likely experienced higher P inputs through animal waste (urine and dung), enriching the soil with both organic and inorganic P forms (Waldrip & Acosta-Martínez, 2014). Moreover, trampling by livestock may have induced soil disturbance and localized aeration, altering redox conditions and destabilizing P-bound compounds, thereby enhancing P release upon rewetting (Lu et al., 2015). Despite these potential contributors, the study’s findings clearly indicate that the variations in P leaching across land uses were mainly governed by the Fe:P molar ratio and the initial extractable P content, as evidenced by the data in Table 1 and Fig. 9.

The observed leaching contents in this study were relatively lower than already reported values (Kaila et al., 2016; Zak & Gelbrecht, 2007; Zak et al., 2010). Likewise, when looking at the release of P at varying rewetting times, a decreasing trend was observed (Fig. 8). This declining trend can be attributed to the resorption of P onto Al and Fe oxides (Wang et al., 2020). The precipitation of vivianite was also observed to lower P contents released into the water (Hoffmann et al., 2012; Rothe et al., 2016). However, the low risk of leaching in our study may be because we simulated the rewetting process in stagnant water with no lateral or vertical movement. However, the cases with higher initial P contents and lower contents of Fe and Al, accompanied by lateral and vertical flow, may pose a higher risk of P leaching during the rewetting process compared to the conditions observed in the current study. Therefore, the studied lowland organic soils under different land uses here can be rewetted with minimal risk of P leaching. These findings can also be useful to the rewetting areas where hydrological flow is limited or can be controlled.

### Multiple factors control P transformation during the rewetting process

Some studies have already been conducted elucidating the influence of rewetting drained peatlands on P transformation into different fractions. For example, Zak and Gelbrecht (2007) collected peat core samples with different degrees of decomposition and evaluated P transformation into different fractions in an incubation study by simulating rewetting at 20 °C. Negassa et al. (2020) collected soil samples from long-term drained and rewetted peatlands (forest, coastal, and percolation mires) and examined P contents in different fractions. However, their findings do not address the fluctuations in internal P transformations during the rewetting process. This is why the current batch incubation study was planned to evaluate the temporal variations in the transformation of P in the soil during the rewetting process for four months at varying temperatures.

**Fig. 10:**
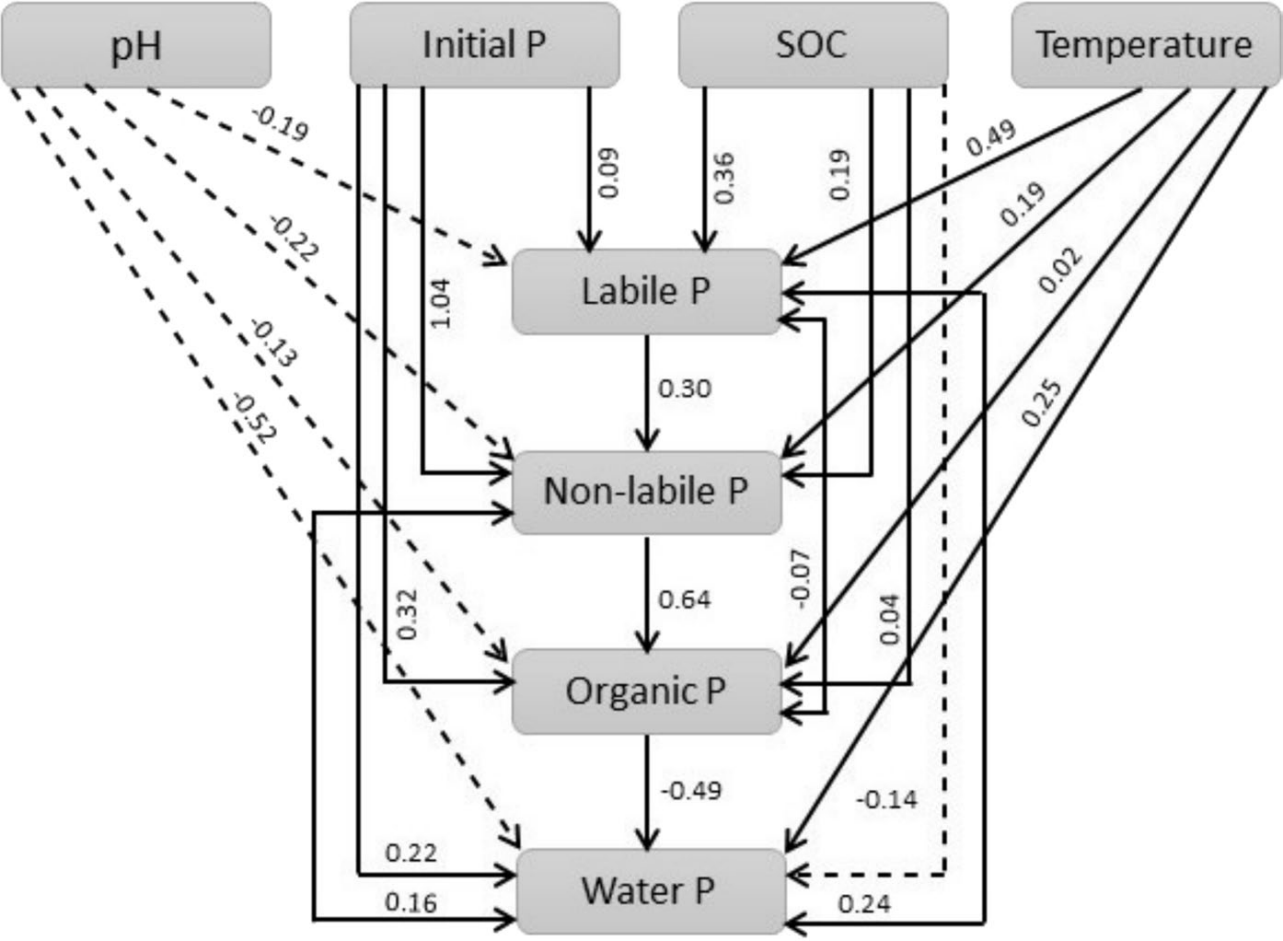
Structure equation modelling effect of pH, initial P, soil organic carbon and temperature effect on different fractions of P

The transformation rate was found to be higher initially and declined with rewetting time (Fig. 10). The initial higher transformation might be attributed to the fast microbial mineralization of organic P compounds into inorganic P (Margalef et al., 2017). Moreover, a high hydrolysis rate of organic P compounds by phosphatase enzymes might also be responsible for a higher initial transformation rate (Park et al., 2022). The lower transformation rates at the later stage of the rewetting process might be ascribed to the declining microbial and enzymatic activities due to the limited availability of readily available organic P compounds (Li et al., 2016).

The process of P transformation and release from organic soil upon rewetting is very complex and the mechanism of its mobility depends on multiple factors including soil organic carbon, initial P contents, iron, and aluminium contents (Kinsman-Costello et al., 2016; Schneider et al., 2019). Structural equation modelling was employed in the current study to evaluate the influence of these factors on P transformation. The positive correlation of initial soil organic carbon with different P pools proves the significant role it plays in P transformation during the rewetting process (Fig. 9). Soil organic carbon influences P transformation in the soil by regulating microbial-driven Fe(III)-oxides reduction and microbial mineralization of organic P (Chen et al., 2019; Hu et al., 2018; Zheng et al., 2019). The intensity of Fe (III) reduction as well as the mineralization of organic P relies on the soil organic carbon as an energy source for microbes and as an electron donor (Maranguit et al., 2017). Likewise, soil organic carbon was also observed to enhance P mobilization and release under anaerobic conditions by stimulating microbial and phosphatase enzymatic activities (Dong et al., 2022; Khan et al., 2022).

The transformation of P is also influenced by the contents of Fe and Al because a large part of the soil P is bound to the oxides of these metals (Lamers et al., 2015). Moreover, both Fe and Al oxyhydroxides received considerable attention because of the significant role they play in binding organic P (Amadou et al., 2022b; Xu et al., 2018). The release of P from these organic P-mineral complexes depends upon the adsorption/desorption capacity as well as microbial activities (Amadou et al., 2022b; Park et al., 2022). The release of *P* from adsorbed glycerophosphate was faster than glucose 6 phosphate and myo-inositol hexakisphosphate depending upon the degree of hydrolysis by enzymatic activity (Amadou et al., 2022a; Annaheim et al., 2010).

### Climate change will promote the potential risk of P leaching

The global climate has undergone significant variations in the last century, characterized by escalating temperatures and an increased occurrence of extreme precipitation events (Masson-Delmotte et al., 2021). Elevated temperatures have the potential to strengthen *P* transformations by accelerating microbial processes and stimulating phosphatase activities (Guo et al., 2021; Li et al., 2019). In this study, a higher transformation rate was observed during rewetting at 20 °C compared with rewetting at 10 °C (Fig. 10).

The transformations of P under two different temperatures explored in this study offer insights into the seasonal variations of potential P leaching during the rewetting process. When to rewet is still an unresolved issue because of seasonal fluctuations over time. Our findings indicate that higher temperatures promote increased P transformation. Therefore, rewetting peatlands during the summer, when soil temperatures are elevated compared to other seasons, could accelerate Fe(III)-oxide reduction and P mobilization (Prem et al., 2015). However, the vigorous uptake of nutrients by vegetation and phytoplankton during the summer months may counterbalance this by reducing net P export from rewetted areas (Pönisch et al., 2023). Additionally, the typically lower precipitation in summer results in stagnant water and reduced hydrological flow, which minimizes *P* transport. Conversely, prolonged water saturation during spring and autumn can lead to extensive dissolution of the entire oxidized Fe pool, increasing the risk of P leaching (Schilling et al., 2019; Smith et al., 2023). Therefore, the timing of rewetting should consider both temperature and precipitation patterns. While our observations may not be universally applicable; however, they are relevant to many regions.

Climate change makes the P leaching scenario more complex because the contents of released *P* increase around 33–41% with incrementing incubation temperature from 10 to 20 °C in this study (Fig. 8). The increase in leaching contents with incremented temperature might be attributed to higher temperature sensitivity of desorption processes than sorption processes (Conant et al., 2011; Hanyabui et al., 2020). Moreover, according to the calculation of temperature sensitivity quotient, the risk of P leaching is expected to increase by 0.24 times (Fig. 7), if we assume a 1.5-degree increase in temperature by 2030 (IPCC, 2021). Hence, there is no doubt that the future temperature rise will boost *P* transformations during the rewetting process, potentially leading to increased P leaching.

Moreover, under climate change scenarios, phosphorus (P) leaching during peatland rewetting can be effectively mitigated through targeted microbial and hydrological management. Maintaining reducing (anoxic) conditions after rewetting helps suppress microbial activity—particularly phosphatase-producing microbes responsible for organic P mineralization (Lin et al., 2020). Strategies such as maintaining high water tables or applying controlled flooding can limit oxygen penetration, thereby restricting aerobic microbial processes that drive *P* release. For instance, Läpikivi et al. (2025) demonstrated the feasibility of using runoff from upstream catchment areas to regulate and sustain elevated water tables in drained/cultivated peatlands in Finland’s western coastal lowlands. Their findings highlight the potential of catchment-scale hydrological management for restoring high water table level in drained agricultural peatlands. Gradual and seasonally timed rewetting—preferably during cooler periods with lower biological activity—can minimize abrupt redox shifts and reduce temperature-driven P mobilization. Additionally, establishing hydrological buffers, such as constructed wetlands or vegetated buffer strips, can intercept and retain leached *P* before it enters nearby water bodies (Walton et al., 2020). Heikkinen et al. (2018) reported that treatment wetlands established on drained peatlands are comparably effective to natural wetland systems in mitigating P leaching. These integrated approaches can help stabilize nutrient dynamics and enhance the ecological resilience of rewetted peatlands under changing climate conditions.

## Conclusions

- The rewetting process triggered a notable P transformation in the soil. The transformation rate was higher initially and declined over time due to the reduction in microbial and enzymatic activity as well as the resorption of the released P.
- The transformation of P from organic soil upon rewetting is a highly complex process, and the mechanism of its mobility is influenced by various factors such as the amount of soil organic carbon, initial P contents, and the presence of iron and aluminium.
- Climate change makes the P transformation scenario more complex by increasing the risk of leaching because of the higher temperature sensitivity of desorption processes than sorption processes.
- Seasonal variations influenced both the transformation and leaching risk of P. Therefore, both temperature and precipitation patterns should be considered for the strategic implementation of the rewetting initiative.
- Future research should explore the intricate interplay between phosphorus (P) transformation processes, seasonal fluctuations, microbial community dynamics, and the diversity of peatland types to develop targeted and sustainable management strategies for P retention during rewetting process.

## Supplementary Information

Below is the link to the electronic supplementary material.

Supplementary file1 (DOCX 380 kb)

## Data Availability

No datasets were generated or analysed during the current study.
