# Supplementary material for: Phosphorus transformations and leaching potential in rewetting drained peatlands: Exploring the influence of land use and temperature
Source: Environ Geochem Health. 2025 Sep 17;47(10):442. doi: 10.1007/s10653-025-02751-y (PMC12443872; doi:10.1007/s10653-025-02751-y)
Supplement: Supplementary file 1 — Supplementary file1 (DOCX 380 kb) [file 10653_2025_2751_MOESM1_ESM.docx]

**Phosphorus Transformations and Leaching Potential in Rewetting Drained Peatlands: Exploring the Influence of Land Use and Temperature**

Atif Muhmood^a,b^, Haonan Guo^a^, Lorenzo Pugliese^a^, Shubiao Wu^a*^

^a^Department of Agroecology, Aarhus University, Blichers Alle 20, 8830 Tjele, Denmark

^b^Institute of Soil Chemistry & Environmental Sciences, AARI, Pakistan

Corresponding author: E-mail address: [wushubiao@agro.au.dk](mailto:wushubiao@agro.au.dk)

**Supporting materials:** 7 pages, 2 Figures, 2 Tables


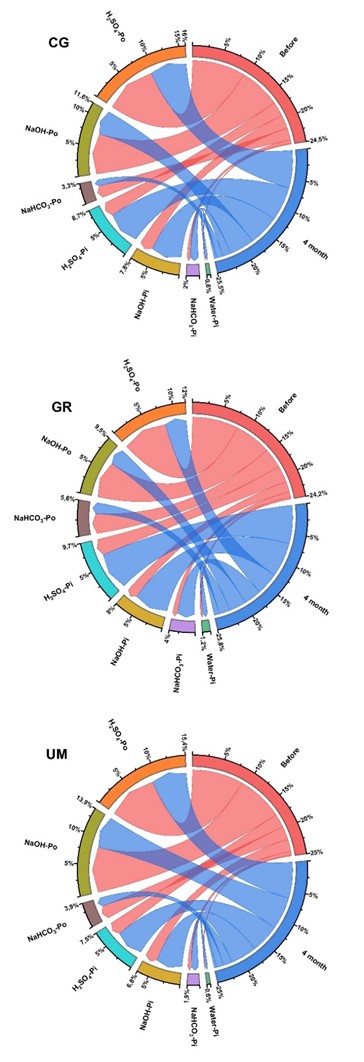


Figure S1. Trend of change in the contents of phosphorus (P) in different fractions in soils under various land uses (CG= cut grass, GR= grazing, UM= unmanaged) after four months of rewetting at 10°C


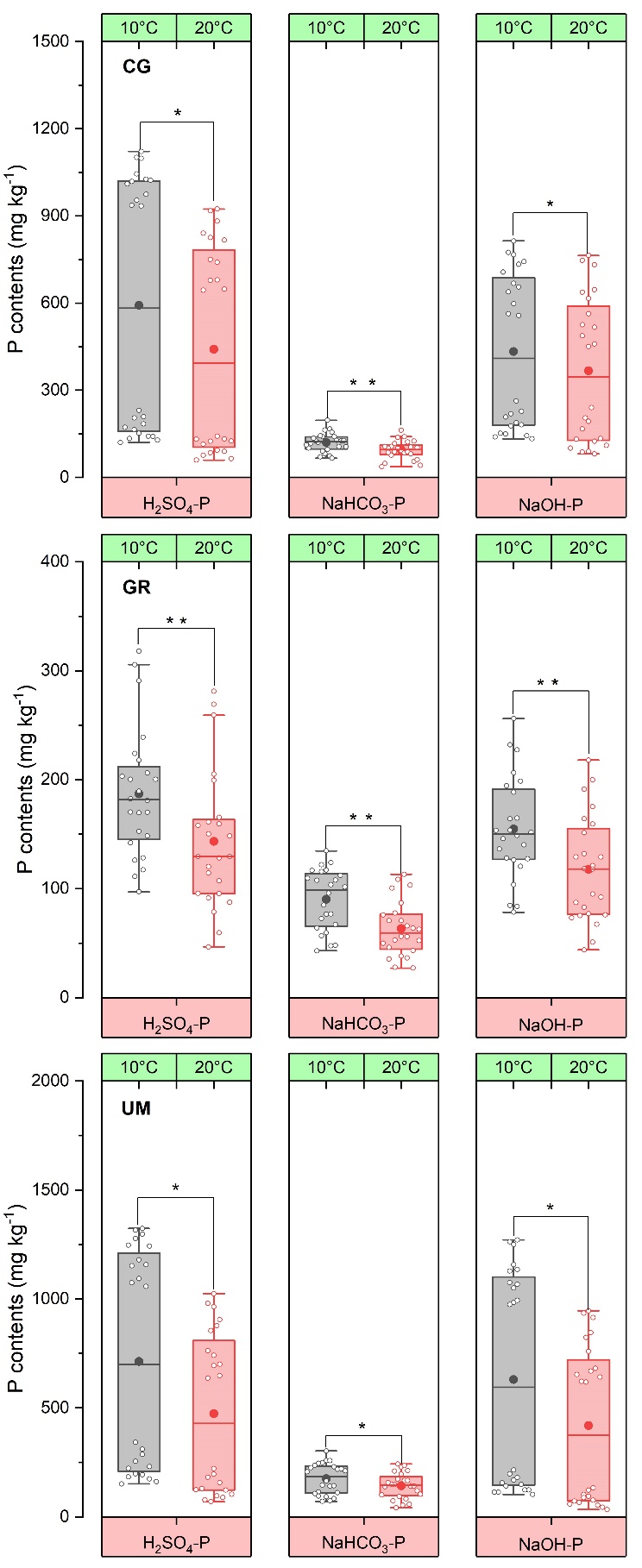


Figure S2. Variation in the contents of organic phosphorus (P_o_) fractions in soils under various land uses (CG=cut grass, GR= grazing, UM=unmanaged) after four months of rewetting under varying temperature

**Table S1. Contents of different P fractions (inorganic and organic) in soils under different land under varying rewetting time at 20 °C**

| Location | Land use | | Time | Water-P_i_ | BD P_i_ | Alkali P_i_ | Acid P_i_ | Total P_i_ | BD P_o_ | Alkali P_o_ | Acid P_o_ | Total P_o_ | Total P |
| --- | --- | --- | --- | --- | --- | --- | --- | --- | --- | --- | --- | --- | --- |
| Skals | CG | 0 | | 10.32 | 57.80 | 237.9 | 256.4 | 562.5 | 218.9 | 869.2 | 1273 | 2362 | 2924 |
|  |  | 1 | | 22.61 | 119.7 | 453.7 | 484.9 | 1086 | 146.8 | 747.5 | 1023 | 1918 | 3004 |
|  |  | 2 | | 28.97 | 138.68 | 562.1 | 594.0 | 1323 | 114.7 | 632.8 | 942 | 1690 | 3013 |
|  |  | 3 | | 35.67 | 144.0 | 647.0 | 683.4 | 1510 | 98.5 | 534.9 | 863.3 | 1496 | 3007 |
|  |  | 4 | | 40.51 | 154.0 | 721.1 | 734.4 | 1647 | 91.0 | 465.6 | 757.0 | 1313 | 2951 |
|  | GR | 0 | | 13.65 | 30.78 | 85.42 | 116.7 | 246.5 | 156.8 | 274.1 | 380.6 | 811.6 | 1058 |
|  |  | 1 | | 21.85 | 78.44 | 189.8 | 210.4 | 500.5 | 108.2 | 202.9 | 269.8 | 581.0 | 1081 |
|  |  | 2 | | 25.38 | 110.2 | 232.1 | 308.5 | 676.2 | 78.33 | 166.2 | 174.7 | 419.3 | 1095 |
|  |  | 3 | | 28.40 | 113.2 | 275.4 | 386.8 | 803.8 | 58.26 | 123.7 | 132.9 | 314.9 | 1118 |
|  |  | 4 | | 33.13 | 121.2 | 319.7 | 419.1 | 893.2 | 46.65 | 84.9 | 105.4 | 237.0 | 1130 |
|  | UM | 0 | | 14.31 | 71.68 | 356.1 | 378.4 | 820.5 | 286.7 | 1344 | 1437 | 3069 | 3889 |
|  |  | 1 | | 28.45 | 115.4 | 493.3 | 574.8 | 1212 | 248.4 | 1122 | 1289 | 2659 | 3871 |
|  |  | 2 | | 33.69 | 132.9 | 591.5 | 765.7 | 1524 | 219.2 | 1019 | 1132 | 2371 | 3894 |
|  |  | 3 | | 37.88 | 143.6 | 674.8 | 868.9 | 1725 | 172.5 | 936.3 | 1033 | 2141 | 3866 |
|  |  | 4 | | 42.14 | 150.9 | 740.5 | 927.9 | 1861 | 152.4 | 855.5 | 961 | 1969 | 3830 |
| Nørre | CG | 0 | | 12.54 | 39.01 | 155.6 | 167.4 | 374.6 | 131.5 | 321.2 | 264.4 | 717.1 | 1092 |
|  |  | 1 | | 16.48 | 68.51 | 234.5 | 254.9 | 574.4 | 106.2 | 212.4 | 178.7 | 497.4 | 1084 |
|  |  | 2 | | 21.62 | 88.14 | 306.7 | 320.2 | 736.7 | 86.6 | 140.9 | 136.8 | 364.4 | 1101 |
|  |  | 3 | | 33.95 | 106.5 | 342.1 | 362.4 | 844.9 | 62.75 | 107.8 | 92.82 | 263.5 | 1108 |
|  |  | 4 | | 38.15 | 121.6 | 355.3 | 382.7 | 897.8 | 46.66 | 92.7 | 86.81 | 226.2 | 1124 |
|  | GR | 0 | | 13.31 | 37.23 | 57.44 | 102.8 | 210.8 | 110.0 | 214.3 | 226.3 | 550.6 | 761.4 |
|  |  | 1 | | 29.14 | 85.18 | 114.1 | 156.7 | 385.2 | 78.27 | 132.6 | 171.5 | 382.5 | 767.8 |
|  |  | 2 | | 34.33 | 118.8 | 182.7 | 207.8 | 543.7 | 56.27 | 98.53 | 126.5 | 281.6 | 825.4 |
|  |  | 3 | | 39.56 | 125.1 | 235.6 | 267.6 | 668.0 | 42.27 | 75.84 | 98.73 | 216.8 | 884.6 |
|  |  | 4 | | 40.90 | 136.2 | 256.7 | 302.7 | 736.5 | 38.27 | 56.84 | 67.26 | 162.3 | 898.8 |
|  | UM | 0 | | 15.43 | 47.87 | 66.72 | 86.99 | 217.0 | 163.7 | 228.2 | 297.6 | 689.6 | 906.6 |
|  |  | 1 | | 36.95 | 97.03 | 115.7 | 155.3 | 404.9 | 145.6 | 162.6 | 233.2 | 541.4 | 946.4 |
|  |  | 2 | | 40.33 | 123.4 | 179.4 | 188.9 | 532.1 | 108.1 | 119.6 | 176.6 | 404.3 | 939.4 |
|  |  | 3 | | 43.29 | 142.4 | 225.4 | 235.3 | 636.4 | 76.46 | 94.35 | 149.4 | 320.1 | 956.5 |
|  |  | 4 | | 45.28 | 151.5 | 241.8 | 260.8 | 699.3 | 67.3 | 74.4 | 109.2 | 251.0 | 950.3 |

Time= months, Water-P_i_= water extractable inorganic P in mg kg^-1^, BD-P_i_ = sodium bicarbonate extractable inorganic P in mg kg^-1^, Alkali-P_i_= NaOH extractable inorganic P in mg kg^-1^, Acid P_i_ = H_2_SO_4_ extractable inorganic P in mg kg^-1^, BD-P_o_ = sodium bicarbonate extractable organic P in mg kg^-1^, Alkali-P_o_= NaOH extractable organic in mg kg^-1^, Acid P_o_ = H_2_SO_4_ extractable organic P in mg kg^-1^

**Table S2. Contents of different P fractions (inorganic and organic) in soils under different land under varying rewetting time at 10 °C**

| Location | Land use | Time | Water-Pi | BD Pi | Alkali Pi | Acid Pi | Total Pi | BD Pi | Alkali Pi | Acid Pi | Total Po | Total P |
| --- | --- | --- | --- | --- | --- | --- | --- | --- | --- | --- | --- | --- |
| Skals | CG | 0 | 10.32 | 57.80 | 237.9 | 256.4 | 562.5 | 218.9 | 869.2 | 1273 | 2362 | 2924 |
|  |  | 1 | 18.76 | 85.52 | 342.3 | 423.2 | 869.7 | 175.2 | 784.7 | 1107 | 2066 | 2936 |
|  |  | 2 | 23.89 | 106.0 | 462.9 | 537.5 | 1130 | 146.2 | 727.5 | 1029 | 1903 | 3034 |
|  |  | 3 | 31.22 | 109.7 | 524.3 | 640.8 | 1306 | 129.4 | 653.8 | 988.6 | 1771 | 3077 |
|  |  | 4 | 34.48 | 119.1 | 566.9 | 682.6 | 1403 | 109.3 | 572.5 | 954.8 | 1636 | 3039 |
|  | GR | 0 | 13.65 | 30.78 | 85.42 | 116.7 | 246.5 | 156.8 | 274.1 | 380.7 | 811.6 | 1058 |
|  |  | 1 | 15.33 | 65.12 | 156.4 | 186.4 | 423.2 | 124.6 | 238.5 | 304.8 | 667.8 | 1091 |
|  |  | 2 | 19.74 | 83.05 | 215.3 | 244.7 | 562.9 | 108.9 | 196.4 | 226.9 | 532.3 | 1095 |
|  |  | 3 | 25.07 | 94.72 | 240.0 | 266.4 | 626.2 | 116.2 | 155.8 | 198.6 | 470.5 | 1096 |
|  |  | 4 | 30.22 | 117.6 | 250.4 | 288.5 | 686.7 | 97.59 | 131.5 | 168.2 | 397.2 | 1084 |
|  | UM | 0 | 14.31 | 71.68 | 356.1 | 378.4 | 820.5 | 286.7 | 1344 | 1438 | 3069 | 3889 |
|  |  | 1 | 18.64 | 86.11 | 461.5 | 494.9 | 1061 | 263.0 | 1261 | 1312 | 2836 | 3897 |
|  |  | 2 | 21.65 | 111.0 | 518.3 | 588.6 | 1239 | 244.4 | 1140 | 1255 | 2639 | 3879 |
|  |  | 3 | 34.31 | 125.4 | 613.9 | 658.7 | 1432 | 226.8 | 1064 | 1163 | 2454 | 3886 |
|  |  | 4 | 38.05 | 129.3 | 685.7 | 726.5 | 1579 | 212.3 | 984.7 | 1085 | 2271 | 3891 |
| Nørre | CG | 0 | 12.54 | 39.01 | 155.62 | 167.4 | 374.6 | 131.5 | 321.2 | 264.4 | 717.1 | 1092 |
|  |  | 1 | 16.47 | 58.51 | 184.5 | 224.8 | 484.3 | 126.2 | 236.4 | 214.7 | 577.4 | 1081 |
|  |  | 2 | 21.62 | 78.14 | 247.7 | 260.2 | 607.7 | 110.6 | 190.9 | 166.8 | 468.3 | 1076 |
|  |  | 3 | 33.95 | 96.48 | 288.0 | 290.4 | 708.9 | 88.73 | 157.8 | 148.9 | 395.5 | 1104 |
|  |  | 4 | 38.15 | 114.6 | 315.2 | 310.7 | 778.8 | 75.66 | 141.1 | 129.8 | 346.5 | 1125 |
|  | GR | 0 | 13.31 | 37.23 | 57.44 | 102.8 | 210.8 | 110.0 | 214.3 | 226.3 | 550.6 | 761.4 |
|  |  | 1 | 23.47 | 58.51 | 94.5 | 143.8 | 320.3 | 86.25 | 165.4 | 184.7 | 436.3 | 756.7 |
|  |  | 2 | 26.62 | 78.14 | 156.7 | 176.2 | 437.7 | 72.5 | 145.9 | 156.8 | 375.4 | 813.2 |
|  |  | 3 | 32.95 | 96.48 | 178.0 | 196.4 | 503.9 | 62.74 | 107.8 | 132.9 | 303.5 | 807.4 |
|  |  | 4 | 36.18 | 114.6 | 205.3 | 220.7 | 576.8 | 52.66 | 96.76 | 123.8 | 273.2 | 850.8 |
|  | UM | 0 | 15.42 | 47.87 | 66.72 | 86.99 | 217.0 | 163.8 | 228.2 | 297.6 | 689.6 | 906.6 |
|  |  | 1 | 20.34 | 76.90 | 104.4 | 135.5 | 337.2 | 150.3 | 186.9 | 259.5 | 596.8 | 934.8 |
|  |  | 2 | 31.30 | 94.47 | 146.0 | 165.8 | 437.6 | 121.2 | 157.1 | 216.2 | 494.6 | 932.2 |
|  |  | 3 | 39.63 | 109.1 | 183.3 | 203.7 | 530.5 | 91.31 | 130.8 | 187.8 | 410.0 | 940.6 |
|  |  | 4 | 45.10 | 116.8 | 206.7 | 246.3 | 610.0 | 84.57 | 113.2 | 145.3 | 343.1 | 954.0 |

Time= months, Water-P_i_= water extractable inorganic P in mg kg^-1^, BD-P_i_ = sodium bicarbonate extractable inorganic P in mg kg^-1^, Alkali-P_i_= NaOH extractable inorganic P in mg kg^-1^, Acid P_i_ = H_2_SO_4_ extractable inorganic P in mg kg^-1^, BD-P_o_ = sodium bicarbonate extractable organic P in mg kg^-1^, Alkali-P_o_= NaOH extractable organic in mg kg^-1^, Acid P_o_ = H_2_SO_4_ extractable organic P in mg kg^-1^
